# Acceptable pregnancy rate of unstimulated intrauterine insemination: a retrospective analysis of 17,830 cycles

**DOI:** 10.1007/s12522-014-0192-2

**Published:** 2014-08-21

**Authors:** Tetsuro Honda, Mai Tsutsumi, Fumio Komoda, Kenichi Tatsumi

**Affiliations:** ^1^ Umegaoka Women's Clinic 1‐33‐3, Umegaoka, Setagaya 154‐0022 Tokyo Japan

**Keywords:** Controlled ovarian hyperstimulation, Intrauterine insemination, Multiple pregnancy, Pregnancy rate, Unstimulated

## Abstract

**Purpose:**

To evaluate the pregnancy rate (PR) of unstimulated intrauterine insemination (IUI).

**Methods:**

This was a retrospective study in a private fertility clinic. Between 2004 and 2013, a total of 4,045 women underwent 17,830 cycles of unstimulated IUI. The etiologies of subfertility in the couples were unexplained (51 %), male factor (36 %), coital problems (9.5 %), and cervical factor (3.5 %).

**Results:**

The PRs/cycle, between the 1st and 9th trials, in women <35, 35–37, 38–40, 41–42, and >42 years of age were 8.2, 7.3, 5.5, 3.6, and 0.9 %, respectively. In 10,076 cycles in which the male partner had a total motile sperm count ≥5 million, the PRs in the respective age groups were 9.9, 8.6, 6.1, 4.8, and 1.2 %. The largest‐sized reported PRs for clomiphene citrate (CC)/IUI in the respective age groups were 11.5, 9.2, 7.3 4.3, and 1.0 % (4,199 cycles in total, Dovey et al., FertilSteril, 2008;90:2281–2286). There were no significant differences in PRs between unstimulated IUI and CC/IUI.

**Conclusion:**

PR for unstimulated IUI was similar to the reported PR for CC/IUI. Although this was a retrospective study without a control group, to reduce multiple pregnancy rate, we believe that unstimulated IUI is a reasonable treatment.

## Introduction

Intrauterine insemination (IUI) is a common treatment for cervical factor, male factor, coital problems, and unexplained subfertility. In a previous review, the pregnancy rate (PR) per cycle for unexplained subfertility was reported to be 1.3–4.1 % with expectant management, 3.8 % with unstimulated IUI, 5.6 % with clomiphene citrate (CC), 8.3 % with CC/IUI, 7.7 % with gonadotropin (GT) administration, 17.1 % with GT/IUI, and 20.7 % with assisted reproductive technology (ART) [1]. With regard to male factor subfertility, PR of GT/IUI is superior to that of unstimulated IUI [2, 3]. Consequently, IUI has been performed generally combined with controlled ovarian hyperstimulation (COH), i.e., with CC and/or GT. The European Society of Human Reproduction and Embryology (ESHRE) Capri Workshop Group reported that PR was 12.4 % in 98,388 cycles of IUI performed in 2004, and that the incidence of multiple births with IUI was 11.8 %, suggesting that most cycles were combined with COH [4]. Since a potential complication of COH/IUI is the increased risk of multiple pregnancies, it was regarded as questionable whether Grade‐A evidence for the efficacy of COH/IUI exists [5]. In addition, van Rumste et al., reported that, in COH/IUI, monofollicular growth is ideal, bifollicular growth is acceptable, but growth of > 2 follicles does not increase PR substantially but increases the multiple pregnancy rate (MPR) [6].

In Japan, as well as worldwide, multiple pregnancies due to subfertility treatment including ART rapidly increased in the 1990s, resulting in a heavy treatment burden in the field of perinatal and neonatal medicine. In 2008, the Japan Society of Obstetrics and Gynecology (JSOG) established a guideline that single embryo transfer (SET) should be done, that double ET could be permitted only in some conditions, and that > 2 embryos cannot be transferred in any conditions [7]. Since then, SET has prevailed in Japan, and in 2011, 75 % (118,179/158,101) of ETs were performed as SETs. In addition, the ratio of ART‐induced multiple pregnancies per ART pregnancies has decreased to 4.2 % [8]. Unfortunately, there are no systematic statistics on the outcomes resulting from non‐ART in Japan, and the JSOG is planning a survey for multiple births resulting from ovulation induction with non‐ART [9]. Although the precise percentage has not yet been made clear, we speculate that multiple pregnancies from non‐ART treatments represent the majority of those resulting from subfertility treatments currently in Japan, especially for high‐order multiples, and that the physicians should make an effort to decrease multiple pregnancies resulting from non‐ART.

Our institution is a private fertility clinic in Tokyo, Japan. Until 2005, for women with ovulatory cycles and unexplained subfertility, we performed 4 cycles of unstimulated IUI, followed by 2 cycles of COH/IUI, and then ART. Since 2006, we have generally discontinued the use of COH/IUI for ovulatory women to decrease the incidence of multiple pregnancies. In the present study, to investigate the efficacy of unstimulated IUI, we analyzed 17,830 cycles of unstimulated IUI. Although retrospective, to our knowledge, this is the largest sample size ever reported.

## Materials and methods

### Our general protocol

For subfertile women with ovulatory cycles and without absolute indication of ART (bilateral tubal obstruction or severe male factor subfertility), if they are aged ≤37 years, we propose 6 cycles of timed intercourse (TI), followed by 6 cycles of IUI, and then ART. For women aged ≥ 38 years, we propose 3 cycles of TI, followed by 3 cycles of IUI, and then ART. Ultimately, however, the final decision regarding the number of cycles of TI and/or IUI performed is made by the couple. Because oocyte donation is not permitted in Japan, we do not limit the upper age for women undergoing IUI. Donor insemination is not performed in our clinic.

### Participants and ethical aspects

We retrospectively analyzed 17,830 cycles of 4,045 women who underwent unstimulated IUI between January 2004 and December 2013.The etiologies of the couples were unexplained (51 %), mild to moderate male factor (36 %), coital problems (erectile disorder, ejaculatory disorder, or dyspareunia; 9.5 %) and cervical factor (3.5 %). Mean age of treated women was 36.6 ± 3.7 years (mean ± SD), ranging from 23 to 52 years. We categorized the women's age as < 35 (1,292 women, 4,443 cycles), 35–37 (1,219 women, 4,794 cycles), 38–40 (999 women, 4,634 cycles), 41–42 (344 women, 2,111 cycles), and > 42 years (191 women, 1,848 cycles). A written informed consent was obtained from each couple. This study was approved by the ethical committee of Japanese Institution for Standardizing Assisted Reproductive Technology(JISART).

### Timing of IUI

To determine the timing of IUI, patients visited our clinic a few days before the estimated ovulation and underwent an ultrasonography scan and assessment of urinary luteinizing hormone (LH) using an assay kit. When a follicle with a diameter of > 18 mm was detected and the urinary LH was positive, IUI was performed the next day.We did not use human chorionic gonadotropin as ovulation trigger routinely.

### Sperm preparation

Sperm was prepared by density‐gradient centrifugation using Sil‐Select Plus^®^ (Ferti Pro N.V., Beemem, Belgium). The volume of the final suspension of motile sperm was 0.5 mL and insemination was performed using a Kitazato IUI catheter^®^ (Kitazato Corporation, Tokyo, Japan).

### Luteal support

Luteal support was not performed routinely but performed only for women whose mid‐luteal progesterone was < 10 ng/mL in the previous cycle. For luteal support, we administered 4 mg/day of chlormadinone acetate (Lutoral^®^, Shionogi Pharmaceutical Co., Osaka, Japan) for 10 days immediately after ovulation.

### Outcomes

“Pregnancy” was defined as clinical pregnancies, including an intrauterine pregnancies in which a gestational sac was confirmed on ultrasonography, and ectopic pregnancies that required surgical/medical intervention. Biochemical pregnancies were not included in “pregnancy.” “Live birth” includes live births and ongoing pregnancies after 11 weeks. Stillbirths were not included in “live birth.”

### Statistics

For statistical analysis, we used a Chi square for independence test. The difference was considered significant at *P* < 0.5.

## Results

**Figure 1:**
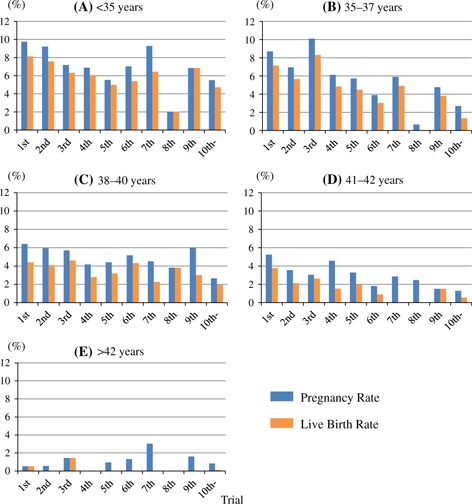
Pregnancy rate (PR) and live birth rate (LBR) per trial cycle in women at <35 (**a**), 35–37 (**b**), 38–40(**c**), 41–42 (**d**), and >42 years of age (**e**)

PR and live birth rate (LBR) per trial in each age group are shown in Fig. 1. The mean PR and LBR per cycle between the 1st and 9th trials were 8.2 and 6.9 % in women at < 35 years (Fig. 1a), 7.3 and 5.9 % at 35–37 years (Fig. 1b), 5.5 and 3.9 % at 38–40 years (Fig. 1c), 3.6 and 2.1 % at 41–42 years (Fig. 1d), and 0.9 and 0.3 % at >42 years (Fig. 1e), respectively.The rate of multiple birth per live birth was 0.6 % (5/780) and all cases were twin pregnancies.

**Figure 2:**
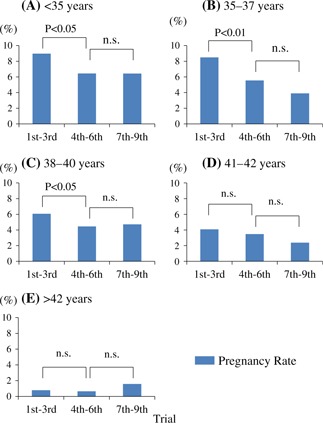
The relation between pregnancy rate (PR) and the number of trial cycle in women at < 35 (**a**), 35–37 (**b**), 38–40 (**c**), 41–42 (**d**), and > 42 years of age (**e**)

To investigate the relation between PR and the trial number, we compared the PR in the 1st to 3rd, 4th to 6th, and 7th to 9th trials (Fig. 2). The PRs were higher in the first 3 cycles, at <35 years (Fig. 2a), at 35–37 years (Fig. 2b), and at 38–40 years (Fig. 2c), but the PRs were not different at 41–42 years (Fig. 2d), and at >42 years (Fig. 2e). The PR in the 4th to 6th trials and that in the 7th to 9th trials were not significantly different in each age group.

**Figure 3:**
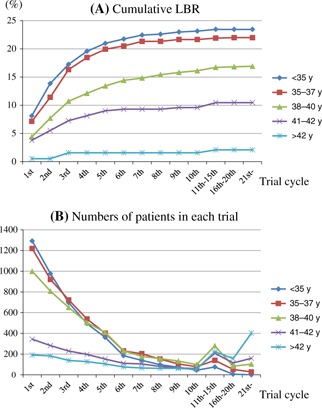
**a** Cumulative live birth rate (LBR) and **b** the number of patients in each trial by age group

The cumulative LBR in each age group is shown in Fig. 3a. The ratio of cumulative LBR in the first 3 cycles per cumulative LBR in the total cycles was 73.6, 74.3, and 66.7 % in at <35 years, at 35–37 years, and at 38–40 years, respectively. The numbers of patients in each trial by age group are shown in Fig. 3b. There were no patients aged <35 years who underwent >15 cycles.

Our primary aim was to compare PR for unstimulated IUI to that for CC/IUI. Dovey et al., reported a retrospective analysis of 4,199 cycles of CC/IUI with total motile sperm count of ≥5 million [10]. We selected the 10,076 cycles in which the male partner had a total motile sperm count ≥ 5 million in the final suspension, and we compared our data to theirs (Table 1). The PRs in the each age group were not significantly different between in our unstimulated IUI and in their CC/IUI.

**Table 1:** Comparison of unstimulated intrauterine insemination (IUI, our data) and clomiphene citrate (CC)/IUI (Dovey et al., 2008, Ref. #10)

	Unstimulated IUI	CC/IUI	Statistics
Author	This study	Dovey et al., Ref. #10	
Study design	Retrospective	Retrospective	
Number of couples	*n* = 2,789	*n* = 1,738	
Number of cycles	*n* = 10,076	*n* = 4,199	
Ovulatory?	Ovulatory	Ovulatory or oligo‐anovulatory	
Total motile sperm count	≥5 million	≥5 million	
Etiology			
Unexplained/endometriosis	Included	Included	
Coital problems	Included	Included	
Cervical factor	Included	Included	
Male factor	Excluded	Excluded	
PR/cycle between the 1st and 9th trials			
≤34 years	9.9 % (2,794 cycles)	11.5 % (2,351 cycles)	n.s. (*P* = 0.069)
35–37 years	8.6 % (2,903 cycles)	9.2 % (947 cycles)	n.s. (*P* = 0.587)
38–40 years	6.1 % (2,711 cycles)	7.3 % (614 cycles)	n.s. (*P* = 0.253)
41–42 years	4.8 % (1,071 cycles)	4.3 % (166 cycles)	n.s. (*P* = 0.757)
≥43 years	1.2 % (597 cycles)	1.0 % (120 cycles)	n.s. (*P* = 0.738)
Multiple pregnancy rate	0.6 % (5/780)	Not mentioned	

## Discussion

As described in the Introduction, the reduction of multiple pregnancies resulting from COH/TI to COH/IUI is speculated to be an important matter currently in Japan. In a review, the twin PR and high‐order multiple PR are 0–28.6 % and 0–9.3 % for high‐dose GT/IUI, 0–29.3 % and 0 % for low‐dose GT/IUI, and 0–12.5 % and 0–3.7 % for CC/IUI, respectively [11]. The rate of multiple gestations for GT/IUI is too high to be accepted currently in Japan. The twin PR for CC/IUI is rather acceptable, but high‐order multiple pregnancies are a problem.

To avoid iatrogenic multiple pregnancies, we performed unstimulated IUI for ovulatory women since 2006 and present a retrospective analysis in this report. Our primary aim was to compare PR for unstimulated IUI to that for CC/IUI. The reported PR per cycle for CC/IUI varies from 2.0 to 19.3 % [11], but the reviewed reports included relatively small sample sizes. Dovey et al., reported a retrospective analysis of 4,199 cycles of CC/IUI with total motile sperm count of ≥ 5 million [10], and we compared our data to theirs (Table 1). The PR/cycle was not significantly different between in our unstimulated IUI and in their CC/IUI. In addition, oligo‐anovulatory women were included in their CC/IUI. There is a report that PR with CC/IUI is higher in anovulatory women than in ovulatory women [12]. Therefore, we consider that PR in our unstimulated IUI was similar to those in their CC/IUI. In a review in 1998, PR with unstimulated IUI was reported to be 3.8 % [1]. This could have been an underestimation. We showed a higher PR with unstimulated IUI in this study. This is probably because fertility clinics are easily accessible in Tokyo, and the patients were able to visit us frequently if necessary, and the appropriate timing of IUI was determined.

Our second aim was to investigate how many cycles of IUI should be attempted. Dovey et al., reported that because the overwhelming majority of pregnancies occur within 3–4 cycles, there seems to be little benefit to continue beyond this [10]. In contrast, Custers et al., reported that PR after the 6th cycle is acceptable [13]. In this study, we showed that PR in the 1st to 3rd trials was higher than that in the 4th to 6th trials in women at < 41 years. Does this suggest that we should recommend the younger women to proceed to ART after the 3rd IUI? However, the ratio of cumulative LBR in the first 3 cycles per cumulative LBR in the total cycles was 67–74 % at <41 years, and; therefore, about 30 % of women were able to have a baby after the 3rd trial. Thus, we consider that the IUI trial after the 3rd is not useless. This issue must be clarified by a randomized controlled study, considering the cost‐effectiveness, because ART has more cost and risk than IUI does. On the other hand, the PR in the 4th to 6th trials and that in the 7th to 9th trials were not significantly different in any age group. So, it seems reasonable to continue IUI after 6th cycle, if the couples request IUI but not ART.

Medico‐social circumstances are different among nations. For reference, the multiple delivery rate per ET was 19.2 % in Europe in 2010 [14]. In the US, the rate of multiple births per all live births resulting from fresh ET from nondonor eggs in women aged < 35 years was 32 %, and that from frozen embryos in women aged < 35 years was 25 % in 2011 [15]. The guidelines for MPRs can vary among nations, and those in other nations would likely not to apply in Japan. Thus, they are not presented in this report. The main conclusion of the present study is that PR in unstimulated IUI is acceptable in the context of strong social needs to decrease iatrogenic multiple births in Japan. In conclusion, we believe that unstimulated IUI has been underestimated and is a reasonable choice of antecedent treatment to ART.

## Acknowledgments

We truly appreciate our embryologists, Yuko Kanai (chief), Yuki Hamasaki, Tomoko Nishikawa, KanakoNamie, Moemi Nobuta, YurieShibao, Marie Higashi, and Yoshiya Iida, for clinical work and recording outcomes.

### Conflict of interest

All the authors state explicitly that there are no conflicts of interest in connection with this article.

### Human rights statements and informed consent

We obtained a written informed consent from each participant couple. This study was approved by the ethical committee of Japanese Institution for Standardizing Assisted Reproductive Technology (JISART).

### Animal studies

Not applicable.
